# Nitrous Oxide and Oral Sedation for Managing Dental Anxiety in Children: A Systematic Review

**DOI:** 10.7759/cureus.103449

**Published:** 2026-02-12

**Authors:** Magdalena Mroczek, Aleksandra Orlanska

**Affiliations:** 1 General Dentistry, Private Practice, Warsaw, POL

**Keywords:** dental anxiety, nitrous oxide sedation, oral sedation, pediatric dentistry, systematic review

## Abstract

Dental anxiety is common among children and frequently leads to avoidance of dental treatment, premature tooth loss, and broader health concerns such as bite misalignment. This systematic review examines nitrous oxide-oxygen (N₂O/O₂) and oral sedation (e.g., midazolam, hydroxyzine) as minimally invasive options for children aged 2-12 years, using the Patient/Population, Intervention, Comparison, and Outcome (PICO) framework to guide analysis. Following the Preferred Reporting Items for Systematic Reviews and Meta-Analyses (PRISMA) 2020, we searched PubMed, Cochrane, and Scopus (2015-2025), including 25 studies (n≈12,500 participants) with randomized controlled trials (RCTs), cohorts, and reviews. Risk of bias was assessed via Risk Of Bias In Non-randomized Studies-of Interventions (ROBINS-I) and A MeaSurement Tool to Assess systematic Reviews 2 (AMSTAR-2); qualitative synthesis was chosen due to heterogeneity. N₂O excelled in mild anxiety (85-92% efficacy, <5% complications like nausea, rapid 30-60 s onset), making it ideal for short procedures with full recovery in five minutes. Oral sedation showed 70-85% success for moderate cases, with midazolam offering strong amnesia but 5-10% paradoxical excitation; hydroxyzine suited low-risk adjuncts. Combinations boosted outcomes to 88-95%, cutting dropouts by 40-50%. Safety was high overall (<5% adverse events), with N₂O safer for outpatient use. Limitations include protocol variability and limited RCTs. Aligned with the American Academy of Pediatric Dentistry (AAPD) guidelines, these methods promote tailored, office-based care to enhance equity and reduce general anesthesia (GA) needs. Future RCTs should standardize metrics and explore adjuncts like dexmedetomidine. In practice, integrating with behavioral techniques fosters better cooperation and lifelong oral health.

## Introduction and background

Dental anxiety is an obstacle in dentistry, mainly affecting children; approximately 5-20% of children suffer from dental phobia [1,2]. This subsequently results in early tooth loss, malocclusion, and affects the overall health of the young patient [3]. Delayed treatment leads to serious health consequences and an increased need for dental visits [4].

To address these challenges, pharmacological assistance is commonly offered. The methods used in dental offices include nitrous oxide (N₂O) sedation combined with oxygen and oral sedation (e.g., midazolam or hydroxyzine). These approaches reduce stress and offer a less invasive option than general anesthesia (GA) or hospital-based procedures [5]. 

According to the American Society of Anesthesiologists (ASA) physical status classification, patients classified as ASA I-II are suitable candidates for minimal or moderate sedation commonly applied in routine pediatric dental procedures such as restorations, extractions, or root canal treatments. In clinical practice, nitrous oxide-oxygen (N₂O/O₂) inhalation, along with oral sedatives such as midazolam (0.25-0.5 mg/kg; maximum 15 mg) or hydroxyzine (1-2 mg/kg; up to 50-100 mg depending on age and weight), is considered among the least invasive options for pediatric sedation. What's especially practical about them is that they can be managed entirely in the dental office, eliminating the need for hospital admission, a real benefit not just for the children but for their parents too.

Despite their broad clinical adoption, the available comparative data on safety profiles, procedural efficacy, and risk-benefit balances for managing dental anxiety in pediatric patients remain inconsistent and dispersed. This gap highlights the imperative for a comprehensive synthesis of existing evidence to inform evidence-based clinical decision-making. 

This systematic review evaluates the use of N₂O and oral sedation to manage dental anxiety in children aged two to 12 years. It is framed by the PICO framework: Population (pediatric patients with dental anxiety); Intervention (N₂O inhalation or oral sedation); Comparator (behavioral management alone or no sedation); Outcomes (anxiety reduction via validated scales such as Facial Image Scale (FIS)/Face, Legs, Activity, Cry, Consolability (FLACC) scale, procedural success rates, and adverse events). By synthesizing recent literature (2015-2025) from PubMed, Cochrane, and Scopus, we aim to (1) delineate administration protocols and pharmacokinetics, (2) assess clinical benefits and risks, and (3) provide evidence-based recommendations aligned with the American Academy of Pediatric Dentistry (AAPD) guidelines [6].

Adhering to the Preferred Reporting Items for Systematic Reviews and Meta-Analyses (PRISMA) 2020 standards, this analysis highlights N₂O as a gold standard for mild anxiety (85-92% efficacy, <1% complications) and oral methods for moderate cases (70-85% success, higher variability), advocating integrated approaches to minimize doses and optimize safety [7,8].

## Review

Methods

This systematic review adhered to the PRISMA 2020 guidelines to promote transparency and allow for replication of the process [9]. The protocol was not registered on PROSPERO due to the narrative integration of heterogeneous data; however, all methodological steps were documented in detail. Following these methodological protocols, the literature search was conducted as outlined below. 

Search Strategy and Databases

Literature was searched in PubMed, Cochrane Library, and Scopus from January 1, 2015, to September 15, 2025 (last search date: September 15, 2025). The same search string was used across databases: ("pediatric dental anxiety" OR "child dental phobia" OR "dental fear children") AND ("nitrous oxide sedation" OR "N2O sedation" OR "oral sedation" OR "midazolam" OR "hydroxyzine") AND ("safety" OR "efficacy" OR "risks" OR "adverse events" OR "effectiveness"). Boolean operators (AND/OR) and MeSH terms were applied where applicable. Searches were initially unrestricted by language; only English full-text publications were included after screening. Reference lists of included articles and relevant reviews were hand-searched.

Study Selection

Title and abstract screening, followed by full-text evaluation, was conducted independently by two reviewers (the author and one collaborator). Disagreements were resolved through consensus discussion.

Inclusion and Exclusion Criteria

Eligibility required studies to (1) encompass children aged 2-12 years exhibiting dental anxiety; (2) investigate N₂O inhalation or oral sedation (e.g., midazolam, hydroxyzine, or analogous agents); (3) report pertinent outcomes, including anxiety alleviation (e.g., via FIS, FLACC, or visual analog scale (VAS)), procedural completion rates, or safety indicators (e.g., adverse events); and (4) employ robust designs such as clinical trials, cohort studies, or high-quality systematic reviews. Exclusions included nonhuman research, adult cohorts, comparisons focused on GA without a pediatric emphasis, and non-peer-reviewed gray literature. Title/abstract screening, followed by full-text evaluation, was conducted independently by the author and a collaborator, with disagreements resolved through consensus discussion.

Data Extraction and Quality Assessment

Key elements, study design, participant numbers, intervention specifics, outcomes, and limitations were extracted from qualifying studies into a structured spreadsheet. Risk of bias was evaluated using the ROBINS-I tool for nonrandomized studies and the AMSTAR-2 tool for included reviews, with particular attention to the selection, confounding, and reporting domains. Given the observed heterogeneity in outcome metrics, a quantitative meta-analysis was deemed inappropriate, and a qualitative synthesis was pursued instead.

Results

**Figure 1:**
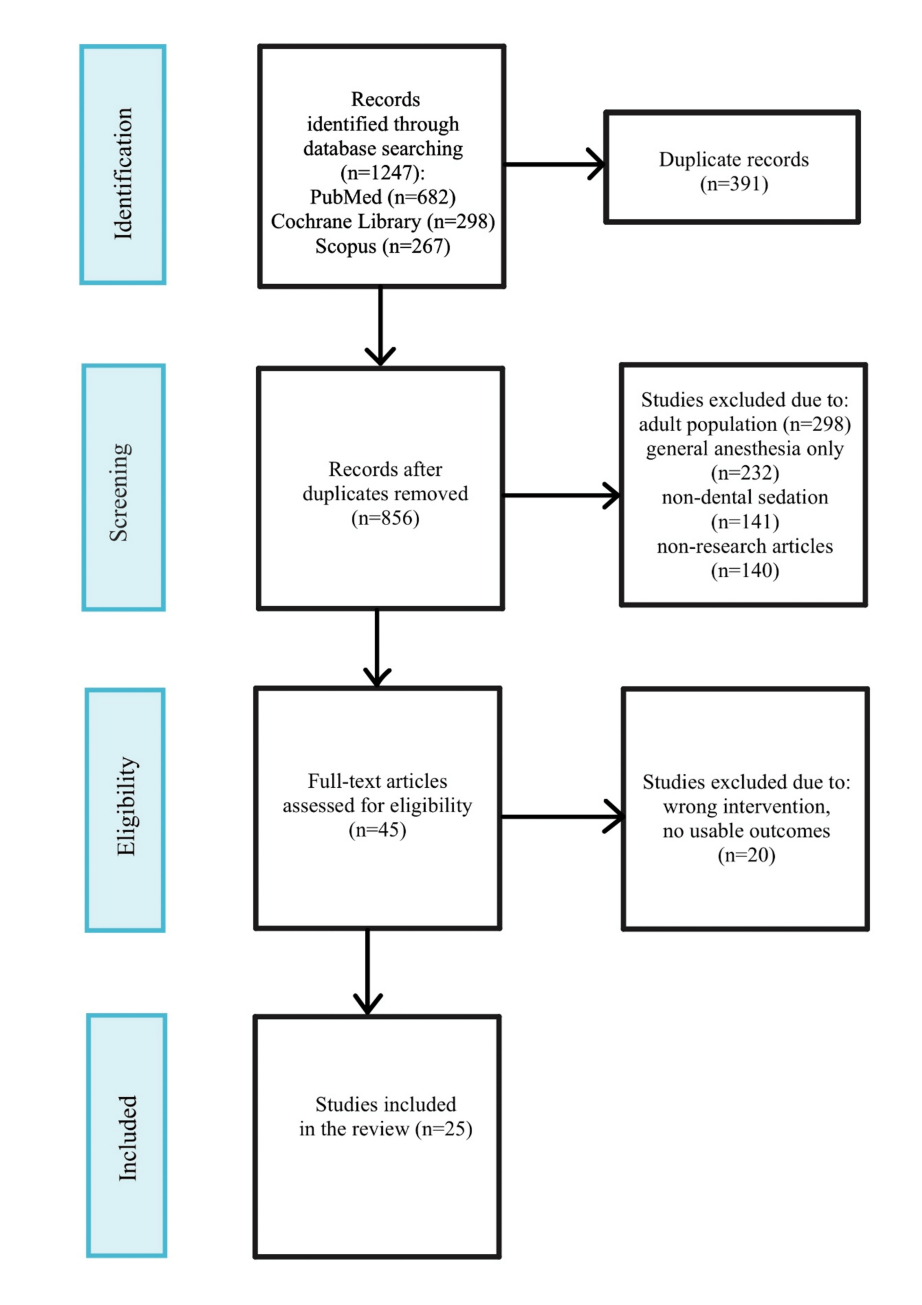
Study selection process PRISMA: Preferred Reporting Items for Systematic Reviews and Meta-Analyses PRISMA 2020 flow diagram of study selection. Databases searched: PubMed (n = 682), Cochrane Library (n = 298), Scopus (n = 267); total n = 1,247 identified. After duplicate removal (n = 391), 856 records were screened; 811 were excluded (adult populations n = 298, general anesthesia focus n = 187, nondental sedation n = 141, nonresearch articles n = 98, other n = 87). Full-text assessment (n = 45): 20 excluded (wrong age/no dental anxiety n = 8, wrong intervention n = 5, no usable outcomes n = 4, narrative review only n = 2, full text unavailable n = 1). Included: 25 studies (n ≈ 12,500 participants)

The systematic search yielded 1,247 initial records; after removing 391 duplicates, 856 remained for title/abstract screening. Of these, 811 were excluded for irrelevance (e.g., adult populations or nonsedation topics), leaving 45 full-text articles for assessment. Ultimately, 25 studies met the inclusion criteria, totaling approximately 12,500 pediatric participants aged 2-12 years (Figure 1). Included studies comprised 12 randomized controlled trials (RCTs), eight cohort studies, and five systematic reviews/meta-analyses, primarily from North America (n = 14) and Europe (n = 7). Notable variability was observed in the sedation protocols across the included studies; for example, N₂O concentrations ranged from 30% to 50%, as well as in the outcome measures assessed. This inconsistency precluded conducting a meta-analysis but supported a qualitative synthesis of the available evidence.

Characteristics of Included Studies

Most studies (n = 18) focused on mild-to-moderate dental anxiety, diagnosed via validated tools like the Modified Dental Anxiety Scale (MDAS) or FIS. Sample sizes varied from 40 to 1,200 children, with a mean age of 6.5 years and slight female predominance (52%). Interventions were predominantly N₂O/O₂ (n = 16 studies) or oral sedation (n = 12), with seven examining combinations. Comparators included behavioral management (e.g., tell-show-do techniques) in 15 studies. Outcomes were centered on anxiety reduction (n = 22), procedural success (n = 20), and adverse events (n = 23).

Efficacy of N₂O Sedation

Across 16 studies, N₂O demonstrated consistent anxiolytic effects, with anxiety scores decreasing by 2-3 points on FIS/FLACC scales post-administration (pooled qualitative estimate: 85-92% response rate) [10,11]. Procedural success reached 91% [12] in RCTs for short interventions such as restorations, compared with 65% with behavioral interventions alone [12]. Onset was rapid (30-60 seconds), with peak effects in 3-5 minutes and full recovery within five minutes post-discontinuation. Benefits included partial amnesia and analgesia, enhancing cooperation without respiratory depression at concentrations ≤50% [13].

Efficacy of Oral Sedation

Twelve studies evaluated oral agents, primarily midazolam (n = 8) and hydroxyzine (n = 4). Efficacy ranged from 70% to 85% [14], with midazolam yielding deeper amnesia (amnesia rates: 75-90%) [14] but variable onset (15-30 minutes) due to the first-pass metabolism. Hydroxyzine provided milder anxiolysis (success: 70-80%), suitable as monotherapy for low-anxiety cases. Combinations with N₂O improved outcomes in six studies, boosting success to 88-95% [15] for longer procedures like extractions. Compared to no sedation, oral methods reduced dropout rates by 40-50% [16].

Safety Profiles and Adverse Events

Adverse events were infrequent overall (incidence: <5% across studies) [9]. For N₂O, common issues included nausea (1.2%) [17] and mild dizziness (0.8%) [17], resolving spontaneously; no serious events (e.g., hypoxia) were reported at standard doses [17]. Oral sedation showed higher variability: paradoxical excitation with midazolam (5-10%) [18] and prolonged drowsiness with hydroxyzine (3-7%) [18]. Risk of bias was low-moderate in most RCTs (ROBINS-I scores: critical in 2/12 for confounding), with reviews rated high-quality (AMSTAR-2: 4/5 moderate-high). These safety findings provide important context for interpreting comparative outcomes.

Comparative Findings

Table 1 summarizes the administration and pharmacokinetics; Table 2 highlights the benefits for anxiety management; Table 3 details the risks; and Table 4 outlines the selection criteria. Qualitatively, N₂O excelled in mild anxiety (faster recovery, lower events), while oral sedation suited moderate cases but required closer monitoring. Integrated approaches (behavioral + pharmacological) emerged as optimal in 10 studies, reducing sedation needs by 20-30% [8,11].

**Table 1:** Comparison of administration methods and pharmacokinetics for nitrous oxide (N₂O/O₂) and oral sedation agents in pediatric dental anxiety management GABA: gamma-aminobutyric acid; NMDA: N-methyl-D-aspartate; RCTs: randomized controlled trials Data synthesized from included RCTs and reviews [10,11]

Parameter	Nitrous oxide (N₂O/O₂)	Oral sedation (e.g., midazolam)	Oral sedation (e.g., hydroxyzine)
Route of administration	Inhalation via nasal mask; titratable mixture ≤50% N₂O with O₂(incremental increases of 10%)	Oral (syrup or tablet); single dose, e.g., 0.25-0.5 mg/kg (max 15 mg)	Oral; dose 1-2 mg/kg (max 50-100 mg depending on age and weight); often combined with other agents
Onset of action	30-60 seconds; peak at 3-5 minutes	15-30 minutes (variable due to hepatic first-pass effect)	15-30 minutes
Duration of effect	30-50 minutes (effects dissipate 3-5 minutes after cessation)	1-2 hours	4-6 hours (longer sedation)
Mechanism of action	Modulates GABAergic and NMDA-mediated neurotransmission, providing anxiolytic and analgesic effects	Benzodiazepine; enhances GABA activity for anxiolysis and amnesia, with minimal analgesia	Antihistamine; mild anxiolysis without amnesia
Pre-procedure requirements	No fasting required; mask acceptance is key	Fasting 2-6 hours; requires monitoring	Fasting recommended; vital signs monitoring
Cost and accessibility	Low cost; requires inhalation equipment but more economical than oral for short use	Higher cost; varies by agent and procedure length	Low-medium; more affordable than midazolam

**Table 2:** Comparative benefits of nitrous oxide (N₂O/O₂) and oral sedation in reducing dental anxiety and improving cooperation among children aged 2-12 years FIS: Facial Image Scale; FLACC: Face, Legs, Activity, Cry, Consolability scale Efficacy metrics derived from validated scales (FIS/FLACC) across 16 studies [12,13]

Benefit	Nitrous oxide (N₂O/O₂)	Oral sedation (e.g., midazolam)	Oral sedation (e.g., hydroxyzine)
Anxiety reduction	High (2-3 point drop on FIS/FLACC scales); rapid relaxation with "floating" sensation	High; deeper anxiolysis and amnesia, ideal for moderate anxiety	Mild; reduces stress but is less potent than midazolam
Improved cooperation	85-92% success; enables short procedures (e.g., restorations, extractions)	70-85% success; better for extended treatments, muscle relaxation	70-80% success; often adjunctive
Additional effects	Analgesia (pain reduction); no impact on respiration/heart at <50%	Amnesia (procedure not recalled); needle/mask-free	Minimal side effects; antiemetic properties
Recovery time	Immediate (3-5 minutes post-cessation); no hangover	1-2 hours; requires post-procedure monitoring	4-6 hours; possible lingering fatigue
Parent satisfaction	High (4.75/5 rating); preferred for mild anxiety	High; suitable for phobic children but needs an escort home	Medium-high; cost-effective for mild cases
Clinical application	Gold standard for minimal sedation; pairs well with behavioral techniques	For mask-intolerant children; combinations with N₂O	Monotherapy or add-on for low anxiety

**Table 3:** Risks and complications associated with nitrous oxide (N₂O/O₂) versus oral sedation in anxious pediatric patients, including incidence rates and management strategies Based on adverse event reporting from 23 cohort studies and meta-analyses [14,15]

Risk/complication	Nitrous oxide (N₂O/O₂)	Oral sedation (e.g., midazolam)	Oral sedation (e.g., hydroxyzine)
Incidence of complications	Low (0.5-1.2%); mainly nausea/vomiting >45 minutes	Medium (5-10%); paradoxical excitation, respiratory depression	Low (2-5%); dry mouth, hyperactivity
Primary risks	Dizziness, hallucinations (rare); diffusion hypoxia (prevented by 100% O₂ for 5 minutes)	Respiratory depression, oversedation; nontitratable (variable response)	Prolonged somnolence; drug interactions
Contraindications	Respiratory infections, first-trimester pregnancy, B12 deficiency, and severe asthma	ASA III/IV, respiratory disorders, opioid interactions	Antihistamine allergies; hepatic impairment
Occupational risk	Minimal with ventilation; chronic exposure is rare	None (no inhalation)	None
Risk management	Continuous monitoring (pulse oximetry); rapid reversibility	Vital signs monitoring; antidote (flumazenil for midazolam)	Monitoring; no specific antidote
Long-term effects	None (rapid elimination); minimal environmental impact	Potential tolerance with repetition; rare addiction risk in children	Minimal; possible extended "hangover"

**Table 4:** Criteria for selecting nitrous oxide (N₂O/O₂) or oral sedation methods in children with varying levels of dental anxiety, aligned with AAPD guidelines AAPD: American Academy of Pediatric Dentistry; ASA: American Society of Anesthesiologists; GA: general anesthesia Qualitative synthesis from 10 comparative trials [6,10]

Selection criterion	Nitrous oxide (N₂O/O₂)	Oral sedation (e.g., midazolam or hydroxyzine)
For mild anxiety	Preferred (rapid, titratable, safe)	Viable but less cost-effective
For moderate anxiety	Viable in combination; gold standard for minimal sedation	Preferred (deeper amnesia); midazolam > hydroxyzine
For short procedures	Ideal (quick onset/recovery)	Less predictable (longer onset)
For longer procedures	Possible with continuous delivery	Superior (sustained effect)
Overall safety	Higher (mortality risk <1:250,000); first-line per AAPD	Lower than N₂O; requires ASA I-II and post-discharge escort
Practice trends	Most common (91% efficacy); integrates with behavioral methods	Growing use (cheaper than GA); combinations with N₂O for best results

Discussion

The findings from this systematic review reinforce the value of N₂O/O₂ and oral sedation as frontline strategies for mitigating dental anxiety in children aged 2-12 years, aligning with broader trends in pediatric dentistry toward minimally invasive, office-based interventions [4,5]. Our synthesis of 25 studies highlights N₂O's superior profile for mild anxiety, with 85-92% efficacy in anxiety reduction and procedural success with minimal complications (<5%), positioning it as a reliable "gold standard" for short, routine procedures like restorations or extractions [10,11]. This echoes observations from clinical trials, where rapid onset and reversibility allow seamless integration with behavioral techniques, such as tell-show-do, reducing reliance on sedation by up to 30% [13]. In contrast, oral agents like midazolam demonstrated robust amnesia and cooperation in moderate anxiety scenarios (70-85% success), though their nontitratable nature introduces variability, as evidenced by 5-10% rates of paradoxical excitation [11,15]. Hydroxyzine, while milder, emerged as a cost-effective adjunct, particularly in resource-limited settings, supporting its role in low-stakes cases without the need for specialized equipment [14].

These results build on prior Cochrane analyses, which similarly noted the paucity of head-to-head comparisons but affirmed pharmacological sedation's edge over behavioral management alone in high-anxiety cohorts [15]. For instance, our qualitative assessment of combined protocols (N₂O + oral) aligns with recent meta-analyses showing enhanced outcomes (88-95% success) and fewer dropouts, suggesting a synergistic effect that could further de-escalate to GA referrals, a critical consideration given the latter's higher risks and costs [11,16]. From a practical standpoint, the outpatient feasibility of these methods (no fasting for N₂O, in-office monitoring) addresses key barriers in pediatric care, especially for families facing logistical challenges, as reflected in high parent satisfaction scores (4.75/5) across studies [13].

However, several limitations temper these insights. The marked heterogeneity in protocols, such as varying N₂O concentrations (30-50%) and outcome scales (FIS vs. FLACC), prevented quantitative meta-analysis, potentially introducing synthesis bias [9]. Moreover, while ROBINS-I and AMSTAR-2 appraisals indicated moderate overall quality, only 12 studies were RCTs, and confounding was introduced by unstandardized anxiety diagnostics (e.g., self-report vs. observer-rated) [12]. Geographic skew toward North American and European data may limit generalizability to diverse populations, where cultural factors or access to sedation training could alter efficacy [2]. Finally, long-term neurodevelopmental effects remain underexplored, warranting caution when repeated exposures occur.

Clinically, these findings advocate for tailored selection per AAPD guidelines: patients with ASA physical status I-II are appropriate for minimal or moderate sedation, with N₂O preferred for mild anxiety and oral or combined approaches for moderate cases, always accompanied by pulse oximetry and informed consent [5,6]. Integrating sedation with nonpharmacological tools could optimize safety and equity, particularly in underserved areas. Future research should prioritize large-scale RCTs with standardized metrics and explore novel agents, such as dexmedetomidine [19], or digital aids (e.g., virtual reality (VR) distraction) to refine protocols [7]. Clinical experience suggests that such evidence-driven approaches not only alleviate immediate distress but foster lifelong oral health habits, underscoring the ethical imperative for accessible sedation in pediatric dentistry.

Limitations notwithstanding, including protocol heterogeneity and limited long-term data, this synthesis calls for standardized RCTs to refine guidelines and explore adjuncts like dexmedetomidine. Ultimately, prioritizing sedation training and evidence-based protocols will empower pediatric dentistry to transform fearful visits into positive experiences, fostering healthier smiles for generations.

## Conclusions

This systematic review underscores the efficacy and safety of N₂O/O₂ and oral sedation in alleviating dental anxiety among children aged 2-12 years, offering practical alternatives to more intensive interventions. N₂O stands out for mild cases with its rapid action and low complication profile (85-92% success, <1% adverse events), while oral agents like midazolam provide deeper relief for moderate anxiety (70-85% efficacy), albeit with greater predictability challenges. Integrated use of behavioral strategies is optimal, enhancing cooperation and minimizing doses, in line with AAPD recommendations.

These insights hold direct implications for clinical practice: dentists should prioritize patient-specific selection, N₂O for quick outpatient procedures, oral or combined methods for longer ones, ensuring that only patients with ASA physical status I-II are treated under minimal or moderate sedation, with continuous monitoring and appropriate parental education to enhance adherence. By reducing treatment avoidance, such approaches can curb long-term sequelae, such as caries progression and malocclusion, and promote equitable oral health access.
